# New Jersey

**Published:** 1897-08

**Authors:** 


### LEGISLATION.

*New Jersey.*— An act to secure the sanitary condition of cow-stables and dairies, and to prevent the feeding of cows and certain cattle with unwholesome food, which has been referred to the Committee on Agriculture and Agricultural College, was introduced to prevent the feeding of garbage or impure or sour foods to such cattle as have been selected for fattening with a view to using them for food, and also to prevent the cattle being kept in an unhealthful or crowded condition. The fine for such misdemeanor to be not less than $50 nor more than $250, which if not paid at once renders the culprit liable to thirty days' imprisonment. An exception is made of ensilage in a properly prepared condition.

**At Nashville, in September, will be the most important gathering to consider tuberculosis, ever held on American soil.**

